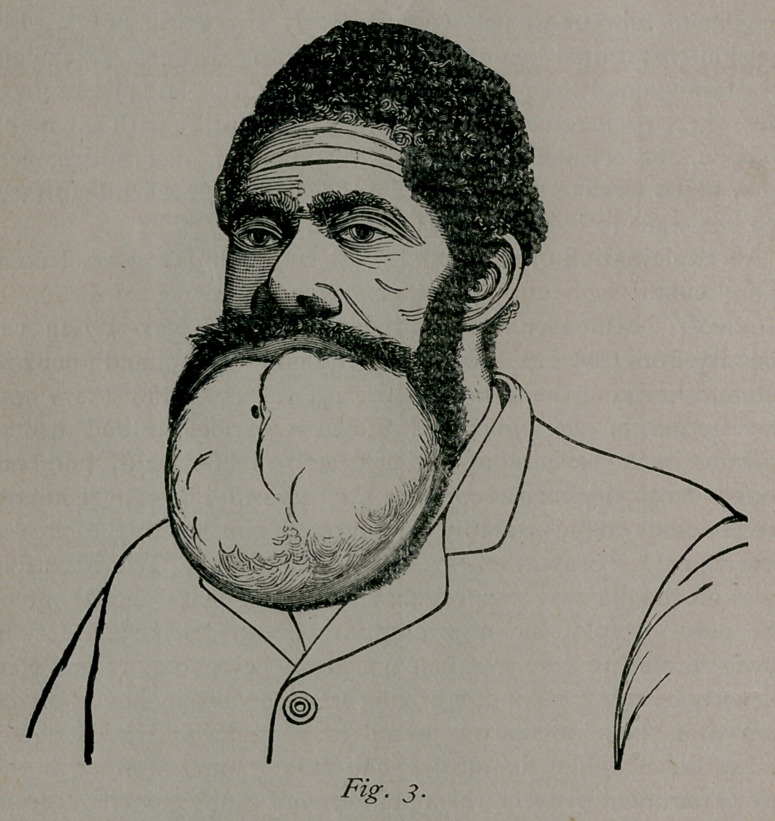# Cystic Tumors of the Inferior Maxilla

**Published:** 1890-09

**Authors:** W. B. Rogers

**Affiliations:** Memphis; Professor Principles and Practice of Surgery and Clinical Surgery, in Memphis Hospital Medical College


DANIEL'S

### Texas Medical Journal.

A Representative Organ of the Medical Profession, and an Exponent of Rational Medicine; devoted to the Organization, Advancement and Elevation of the Profession in Texas.

PUBLISHED MONTHLY.—SUBSCRIPTION $2.00 A YEAR.

Vol. VI. AUSTIN, SEPTEMBER, 1890. No. 3.

### Original Articles.

CONTRIBUTED EXCLUSIVELY TO THIS JOURNAL.

*The Articles in this Department are accepted and published with the understanding that we are not responsible for, nor do we indorse the views and opinions of the writers by so doing.*

For Daniel's Texas Medical Journal.

### CYSTIC TUMORS OF THE INFERIOR MAXILLA.

BY W. B. ROGERS, M. D.,

Professor Principles and Practice of Surgery and Clinical Surgery, in Memphis Hospital Medical College.

EXCLUDING cysts occurring secondarily in tumors found in this bone, the inferior maxilla presents us two varieties of cystic growths: first, cysts occurring in connection with teeth; second, cysts not connected with the teeth and finding origin in the bone proper. Cysts in connection with the teeth are,

[This article appears simultaneously in Memphis Journal Medical Science. —ED.]

1st—polyp like, attached to the roots of "cut" or fully developed teeth.

2d—dentigerous cysts, dependent upon the presence of "uncut" teeth.

It would seem not an uncommon occurrence among dental surgeons of experience, to find small cysts attached to roots of teeth after extraction. These cysts contain a mucus-like substance, and sometimes pus. When small, their very existence is not susceptible of being diagnosed prior to extraction of the teeth; occasionally, however, one of these cysts so enlarges as to form a veritable maxillary growth demanding treatment. Even in such instances, as the tumor requiring the attention of a surgeon, there is no point diagnostic between this and other cysts occurring in this bone. It is only by exclusion, and after incision and exploration, that the nature of such a cyst can be determined.

According to Mr. Heath, these cysts lie beneath the periosteum of the tooth fang; but no hint of the cause of origin have I been able to find.

### DENTIGEROUS CYSTS

Are cysts occurring in the *maxillary bones*, and depend for their origin and existence upon an "uncut" tooth—one that has failed to present its crown through the mucous covering of the gum, by reason of having taken primarily an abnormal direction, or, having primarily been too deep placed in the bone, or lastly, because of a want of development in the fang. This "uncut" tooth (or mayhaps teeth), is always to be found in the dentigerous cyst, which contains besides the tooth a fluid of serous nature, but which may sometimes be mixed with pus by reason of inflammatory changes in the cyst wall. The cyst wall is a tough, fibrous membrane, lined by epithelium, and is natural to the part. It is the sac in which the tooth grows until it reaches the surface of the gum. This sac, by reason of irritation from the retained tooth, becomes hypertrophied, there is an increase of secretion from its epithelial-lined surface, and thus we have a tumor—a cyst due to effusion, differing from the variety of cystic growths which we come next to consider, and which are adventitious cysts.

### CYSTIC TUMORS.

This term, without a qualifying adjective, seems by common consent to be applied to that class of tumors occurring in the maxillary bones and composed of sometimes a single cyst, and at other times many cysts, finding origin in the substance of the bone and not dependent upon any aberration in dentition. They are not cysts due to retention, nor are they cysts dependent on effusion into a sac natural to the part, but are purely new-formed, adventitious cysts. I find several surgical writers describing this affection as cystic disease of the bone because of the fact that the entire body to the bone, and even the rami, may be affected or included in one of these multilocular cysts.

The disease begins at some one point and gradually extends, in some instances throughout the bone; in others it confines itself to a segment which continues to grow while the remaining bone shows no tendency to become affected. It has been my province to remove three of these multilocular cysts, to which Gross applies the term "compound cystic tumor," to distinguish from those composed of a single sac. As my observation goes, these tumors occur later in life than do dentigerous cysts; they are of slow growth, and show little if any tendency to impair the health. In one instance I saw glandular enlargement in the submaxillary region, and there were several loculi of the tumor filled with brain-like matter. I rather feared encephaloid disease had sprung up in the growth, and that it would show itself soon in the enlarged glands, but three years after removal of nearly half the lower jaw (with tumor), the patient was in the best of health, with no evidence of malignant disease.

A section of one of these tumors will show the body of the bone expanded, the internal and external plates of compact tissue greatly thinned and crackling like parchment under palpation; the cancellous tissue will have disappeared, and its place be occupied by a single large cavity or fibrous sac, containing fluid, serous in character, or if it be a multilocular cystic growth, there will be many partitions giving many loculi lined by fibrous, epithelial-covered membranes. The fluid of these sacs varies from clear, egg albumen to dirty brown and even black, blood-stained, watery fluid—and again it is thick and encephaloid-like.

DIAGNOSIS.

Cystic tumors of the inferior maxilla are by no means of rare occurrence, and whatever be their several sources of origin, they present some features in common, namely: slowness in growth, absence of severe pain, absence of glandular involvement, with no constitutional impairment except as attends on any tumor interfering mechanically with mastication and deglutination. There need be little or no difficulty in deciding on the cystic nature of these tumors. The exploring needle will detect the cavity.

1st. Cysts attached to *roots of teeth* can only be diagnosed *in situ* after incision, and are of extremely rare surgical importance.

2d. Dentigerous cysts usually attend on second dentition, rarely in connection with milk teeth, and still more infrequently have they been found with supernumerary teeth. It is only in the comparative early stages of growth of a dentigerous cyst, before it has by increase of size involved the sites of many teeth, besides invading the body of the bone, before the patient's memory has become clouded as to the exact point at which "the swelling was first noticed," before all normal landmarks have changed or passed away, that a diagnosis is at all easily arrived at. Later than this, when the tumor is large and definite information concerning missing teeth is unobtainable, there are few if any points of diagnostic value; nothing short of incision will tell the nature of the tumor. The leading point in a positive diagnosis is the fact that some one tooth (or teeth) which should be present, and that, too, at the site of the tumor, has never shown itself.

3d. Cystic tumor, simple or compound. Instead of the continued non-appearance of some tooth, we usually find that one or two aching permanent teeth preceded the appearance of this tumor, and to the irritation caused by presence of these bad teeth the growth is usually attributed, and probably not unjustly. A multilocular cyst ought readily enough be determined from cysts of any other nature.

TREATMENT.

Obviously, there is but one treatment for these tumors, one and all, and that is surgical. When they have not reached such dimensions as to have completely destroyed all outline and framework of the bone, a free incision, evacuating the contents and packing the sac with gauze, with a view to having same fill by granulation, is the proper treatment, and usually succeeds. In some instances the whole external wall may be cut away and when a multilocular cyst, the contents curetted or gouged out, leaving the internal plate. The incisions in such cases are

*Fig. 1.*

always within the mouth, leaving no defacing cicatrices, and when the inner plate or the continuous line of the body of the bone can be left, there usually follows but slight deformity. There will come up, occasionally, cases in which the tumor has reached such dimensions and played such havoc with the bone, especially compound cystic tumors, that excisions of sections of the bone, or even the entire maxilla, is the proper thing to do. Most of the cases that have come to me for treatment have been of the latter kind, requiring removal of parts of the bone with the tumor—excision.

As an example of a simple cyst of the jaw, I beg to report the following case—illustrated by Fig. 1:

*Case I.* Mrs. K., white, aged 43 years, resident of Mississippi, was referred to me by Dr. W. P. Connor, and presented a tumor involving the right half of the inferior maxilla. The growth began some six years previous to my examination, and by gradual increase had attained the dimensions of an average-sized cocoanut. On one or two occasions it had been opened with a bistoury and contents of a glairy nature evacuated, only to re-accumulate. Besides the disfigurement shown in the accompanying cut, Fig. 1, the growth so filled the buccal cavity as to entirely prevent mastication of food, and it was with great difficulty that the patient could swallow liquid nourishment. As a consequence she was so weakened and emaciated that removal of the tumor could not be advised just then. There was no pain felt in the growth; no tendency to ulceration; no glandular involvement, although so long time in existence; manipulation was painless. Fluctuation was distinct, and while there were many soft points to be detected both on the cutaneous and mucous surfaces, distinct areas of shell-like bone could be mapped out and a parchment-like crackle was very perceptible on palpation. The growth involved three-fourths of the body and lower third of ramus, right side. Both portions of the bone were expanded to a mere shell. The diagnosis of cystic tumor involving the body of the bone was readily enough made, and removal of the bone clearly indicated. It is to be remembered that a free incision, evacuation and packing the cavity is the recommended operation for these tumors, especially those of simple character (single cyst), but in this case there was no outline of body of bone left—only islands of bone scattered irregularly over a thickened sac. Hence, to my mind, a more thorough operation of romoval was indicated. The general health of the patient was so poor that my first step was to evacuate the contents of the tumor by means of an aspirator entered through the buccal surface. More than a pint of chocolate-colored, glairy fluid was drawn off, and the patient thus enabled to take nourishment liberally. At end of three weeks she returned considerably improved, and the tumor had refilled.

An incision was made along the base of the tumor, the facial artery was ligated, and by keeping carefully against the surface of the tumor very little blood was lost. Almost the entire right half of the maxilla was removed. The wound was packed with gauze and ample room left for drainage. Her recovery was rapid and at this time, eight years since operation, she is enjoying excellent health. A gristly mass has taken the place of the bone removed, and very little disfigurement exists.

The tumor proved a simple cyst—the fibrous wall was more than a line in thickness. At the floor of the sac there were several bony partitions projecting half an inch into the cavity. (This specimen is now in the museum of the Memphis Hospital Medical College.)

Illustrative of compound or multilocular cysts of this bone, I beg leave to briefly report three cases:

*Case I.* Patient, a colored woman from Mississippi, 40 years of age, with the usual history of repeated severe and prolonged spells of toothache, preceding the appearance of a swelling in the left half of the lower jaw, which soon developed to a veritable tumor. Upon examination I found her as shown in Fig. 2—a growth as large as a cocoanut. The body of the bone and lower portions of ramus of left side were extended to a thickness of over five inches. There were numerous soft spots yielding a sense of fluctuation between the patches of boue, so that a diagnosis of compound cyst tumor was readily made. Mastication was very much interfered with, but she was able to keep in a good state of flesh on liquid and soft diet. A small exploring trocar revealed several cysts, with as many varieties of fluid. There was no glandular involvement. The indications were that tumor consisted of very many cysts, and consequently much solid matter in shape of partition walls and living membranes. I decided on excision instead of incision and curetting.

My incision was carried from just below the temporo-maxillary articulation, along the base of the tumor to the symphasis, then upward through the lower lip. Hemorrhage was controlled by

*Fig. 2.*

COMP. CYSTIC TUMOR.

forceps. The tumor was then denuded of its coverings, both externally and for the greater part on its internal surface by using the handle of the knife. The bone was cut one-half inch to left of symphasis. The ramus cut through just below the base of coracoid process, which together with condyle was left in position. The remaining attachments of soft tissues to inner aspect were severed and mass removed. Paquelin's cautery was used to touch several bleeding points. The opening in the buccal mucous membrane was closed and nearly two-thirds of the skin incision was united by suture. One opening was left dependent for drainage and the cavity packed with gauze. In spite of great care, there occurred sacculation of pus, in contact with sawn end of bone, and erysipelas supervened, spreading over entire face. After eight weeks all had healed save small superficial surface, and she went to her home. (College Clinic, '89-90.)

An examination of the tumor confirmed the diagnosis of compound cyst.

*Case II.* Multilocular cyst of right half of lower jaw in patient twenty-eight years of age. Was preceded by tooth-ache of much severity; general health of patient poor, and there was some glandular enlargement in nearest cervical glands. The operation was very similar to that described in Case I, and the tumor identical in make-up, excepting two of the cells composing the mass contained brain-like matter, causing me to fear a return of the disease. This patient made a rapid recovery, and three years later was in excellent health. The size of this tumor was almost equal to a large orange—extended from near symphasis to the angle. Paquelin's cautery was found very convenient in checking the free oozing from patches of small vessels. (College Clinic, '88-89.)

*Case III.* Illustrative of cystic disease of the lower jaw is the case of Mr. Henry, forty years of age, very much emaciated from starvation, due to inability to take food. No glandular implication. Nearly all of the bone involved in the tumor. The disease began fifteen years prior lo appearance shown in cut, as small double cyst at roots of incisor teeth. These cysts were opened and injected repeatedly with iodine, nit. silver, etc., but with no benefit. Other cysts began forming. He declined further treatment at that time, and did not consult a physician until February, 1886, when I saw him and advised excision of the entire mass. Operation was performed without encountering much difficulty and but little hemorrhage. All of bone was removed save half of right and the third of left rami. Tongue was kept forward by means of ligature for forty-eight hours, at the end of which time he could control it without assistance. Took nourishment well, and promised fair to recover. On seventh day after

*Fig. 3.*

operation, after sitting up greater part of two days, he had chill and died of congestion. The tumor weighed 7¼ pounds. Transverse diameter was 9½ inches, ant-post. thickness 7¾ in median line. A section showed dozens of cysts, typical of these tumors.

---

The Italian government has ordered that only medical men shall henceforth be entitled to practice dentistry and blood-letting, an order which will interfere with a large number of quacks, and raise dentistry to so high an attitude that its disciples can no longer hesitate as to whether they belong to a trade or a profession.—*Gaillard's Medical Journal.*

## Figures and Tables

**Fig. 1. f1:**
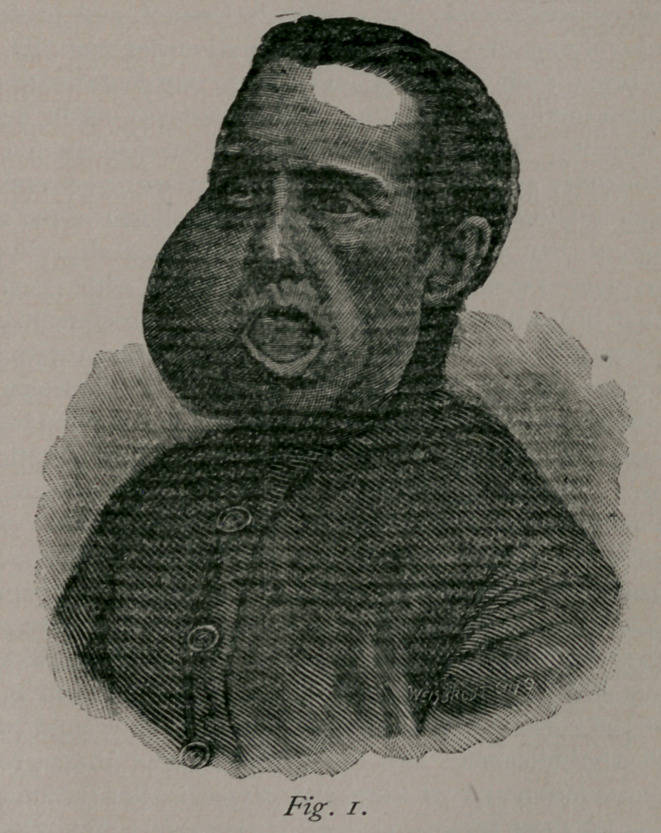


**Fig. 2. f2:**
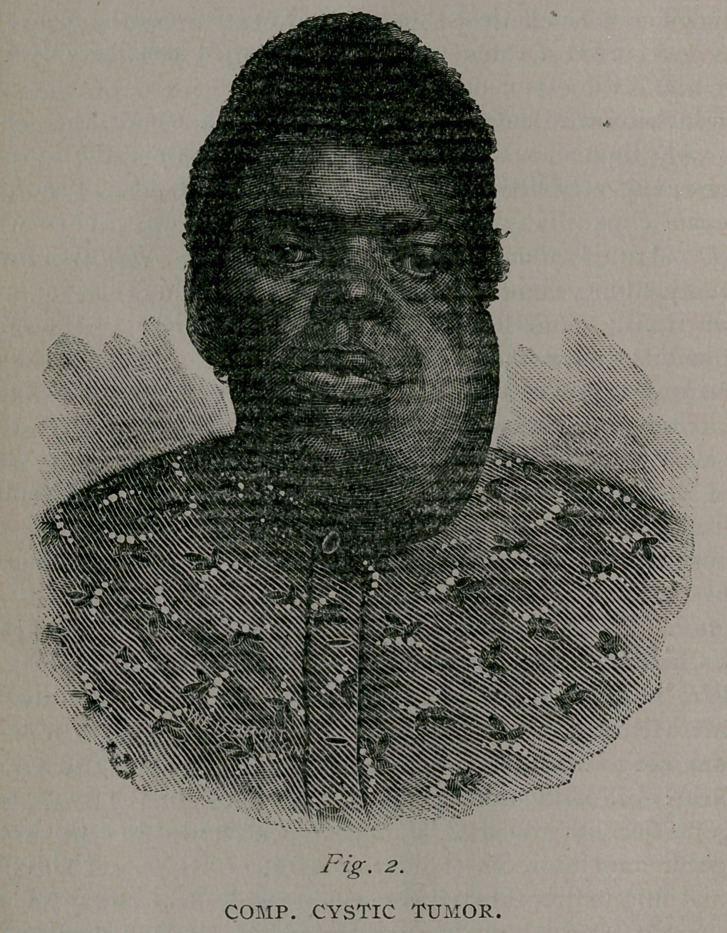


**Fig. 3. f3:**